# Measurement invariance of the Pandemic Anxiety Inventory in different demographic groups

**DOI:** 10.1186/s40359-024-01829-z

**Published:** 2024-06-17

**Authors:** Irvin Sam Schonfeld, Tasmyn Prytherch, Mark Cropley, Jay Verkuilen, Renzo Bianchi

**Affiliations:** 1 Dept. of Psychology, The City College and the Graduate Center of the City University of New York, New York City, USA; 2 The Program in Educational Psychology, the Graduate Center of the City University of New York, New York City, USA; 3 Dept. of Psychology, University of Surrey, Guildford, Surrey UK; 4 Dept. of Psychology, Norwegian University of Science and Technology (NTNU), Trondheim, Norway

**Keywords:** Pandemic Anxiety Inventory, Measurement invariance, Demographic groups, Confirmatory factor analysis, MIMIC analysis

## Abstract

**Background:**

The Pandemic Anxiety Inventory (PAI) was developed in the context of the COVID-19 pandemic. Its content allows it to assess anxiety in connection to any pandemic. Previous research has demonstrated the instrument’s reliability and validity. An important question for clinicians and researchers, however, remains open: Does the PAI have similar meaning for members of different demographic groups? The finding of measurement invariance would allow clinicians and researchers to comparatively assess pandemic-related anxiety across demographic groups, including favored and disfavored groups.

**Methods:**

We conducted a multi-group confirmatory factor analysis to assess the measurement invariance of the PAI using data obtained from a sample of 379 residents of the United Kingdom.

**Results:**

The PAI demonstrated invariance across genders, age groups, individuals who are married or in a relationship and those who are not, as well as individuals with higher and lower incomes. In an ancillary analysis, we found invariance across subsamples of Whites and Nonwhites, although we note that the Nonwhite group was small (*n* = 60) and heterogeneous. The findings of a supplemental MIMIC analysis were consistent with the above.

**Conclusions:**

The PAI shows measurement invariance across a variety of demographic groups. Our findings suggest that the instrument can be meaningfully employed to compare pandemic-related anxiety across these groups.

**Supplementary Information:**

The online version contains supplementary material available at 10.1186/s40359-024-01829-z.

## Background

The Pandemic Anxiety Inventory (PAI) is a 10-item, unidimensional measure developed to assess anxiety symptoms that individuals expressly attribute to the presence of a pandemic [1]. The instrument thus contrasts with cause-neutral anxiety symptom scales such as the Generalized Anxiety Disorder Scale (GAD-7) [2]. While the COVID-19 pandemic has been linked to higher rates of depressive symptoms [3–5], it has also been linked to anxiety [6]. Although the PAI was developed during the COVID-19 pandemic, the instrument was designed such that it can be used in the context of any pandemic.

Research on the PAI indicates that higher scores are related to subjective beliefs about how widespread the COVID-19 virus is in the individual’s locality, reduced social support, financial strain, job insecurity, job loss, sleep problems, stress symptoms, and hospitalization and death of someone close [1]. The PAI exhibits factorial validity and can be used based on its total score. The instrument has also shown evidence of convergent and discriminant validity with regard to a cause-neutral measure of anxiety [1].

Jafari et al. [7] noted that "since clinical decisions about psychological interventions are frequently made on the basis of the results of psychological assessment tools, it is necessary to know whether these instruments function similarly across people with different backgrounds" (p. 120). To make comparisons across demographic groups for clinical *or* research purposes, an instrument must have equivalent meaning across those groups [8–10]. Putnick and Bornstein [11] provided an instructive example of the importance of measurement invariance. They considered a hypothetical scale used to measure the latent construct depression:

- “Suppose frequency of crying, weight gain, and feelings of hopelessness are indicative of the severity of depression in women, but only feelings of hopelessness are indicative of the severity of depression in men. If the three indicators are combined into a scale to compare depression in women and men, mean differences on the scale may mislead because crying and weight gain have little relation to depression in men. In this example, men may score lower than women on the depression scale because they cry less and gain less weight. However, crying and weight gain are not associated with depression in men in the first place” (p. 72).

Measurement invariance across demographic groups is equally important to scales that assess anxiety. Several studies have found evidence of invariance across demographic groups in symptom measures in which anxiety items make up a part of the scale. A few of these studies [7, 12–14] examined invariance in the 21-item version of the Depression Anxiety and Stress Scale, a measure containing seven anxiety items [15]. Two studies found evidence of invariance across groups in the 90- [16] and 62-item [10] versions of the Mood and Anxiety Symptom Questionnaire, an instrument that assesses a combination of anxiety, depression, and general distress symptoms [17]. None of these studies looked specifically at invariance in the anxiety subscales.

There has also been invariance research on several stand-alone anxiety measures, one of which is a measure specifically aimed at excessive worry. Nuevo et al. [18] studied invariance in the 8-item version of the Penn State Worry Questionnaire [19] across samples of Americans and Spaniards who were age 55 and older. The study team found that within women but not men, the scale worked similarly in the United States and Spain. Ober et al. [20], in a study of a newly developed measure of trait test anxiety in U.S. undergraduates, found evidence of scalar invariance across gender, parental educational attainment, and race/ethnicity, although the Nonwhite group was very heterogeneous.

The GAD-7 is an important instrument in research and practice [2]. Moreno et al. [21], in a study of Spanish patients in treatment for emotional disorder, found that a computerized version of the instrument showed evidence of invariance across gender, age, marital status, educational level, employment status (full-time, part-time, unemployed), and time (3 months). In a multi-wave longitudinal study conducted in England, the GAD-7 showed evidence of temporal measurement invariance [22]. Like the study by Moreno et al. [21], the study by Stochl et al. [22] focused on a clinical, not a general population, sample. The GAD-7 is an instrument that is closest to the PAI in terms of symptom coverage.

There has also been research on invariance in COVID-related anxiety scales. Lee [23], using a U.S. sample, showed evidence, although not detailed, of invariance of the Coronavirus Anxiety Scale (CAS) across age, gender, and race. In an ambitious cross-national study of the 7-item Fear of COVID-19 Scale (FCV-19S), Sawicki et al. [24] found that unidimensionality could not be observed without ad hoc modifications. The FCV-19S showed evidence of partial scalar invariance for gender (after relaxing intercept fit for some items) and educational level (after relaxing intercept fit). The PAI has little item overlap with the CAS and FCV-19S; unlike those two measures, the PAI’s items derive directly from the *DSM-5* symptoms for generalized anxiety disorder [25].

The purpose of the current study is to determine if the PAI measures the same construct, namely, pandemic-related anxiety, across different demographic groups. Chan [26] underlined the importance of invariance to the validity of research findings:

The validity of these inferences is dependent on the often untested assumption that, across groups, the same items or scales are measuring the same construct and measuring it with the same precision. When this assumption of measurement invariance is in fact violated, absolute differences in scores between groups, and therefore inferences based on these differences, are likely to be misleading or not meaningful. Hence, measurement invariance is often a statistical hurdle that should be cleared before making direct between-group comparisons of scores (p. 108).

We assessed, using multi-group confirmatory factor analysis (CFA) [27], the measurement invariance of the PAI across genders, age groups, and relationship statuses. We also assessed the extent to which the PAI has similar meaning in favored and disfavored groups by examining invariance by income and race/ethnicity. We evaluated the PAI for (a) configural invariance, (b) metric invariance, and (c) scalar invariance. Configural invariance reflects the extent to which the fit of the overall factorial structure applies across groups; metric invariance assumes configural invariance and reflects the extent to which the factor loadings could be viewed as equivalent across groups; and scalar invariance assumes metric invariance and reflects the extent to which item thresholds are equivalent across groups [11]. If measurement invariance were found, those results would build confidence among clinicians and researchers in PAI-based assessments of meaningful differences in pandemic-related anxiety in patients and research participants.

## Methods

### Sample

In this psychometrically-driven extension of the paper by Schonfeld et al. [1], we originally recruited 424 participants living in the United Kingdom but excluded (a) 28 because they did not respond to the PAI items, (b) nine because they responded affirmatively to a filter question that asked if they responded randomly, and (c) another eight individuals who failed to respond to the item asking about random responding. The final sample thus comprised 379 adults (age > 18). The mean age was 33.21 (*SD* = 12.24). Median income was ₤50,000–₤54,000 (interquartile range from ₤30,000–₤34,00 to ₤70,000–₤74,00). Other demographic characteristics of the sample are presented in Table 1. Participants were well-educated, with 97% having at least some college or university education.

**Table 1:** Summary of demographic variables

	*n* (%)
Males	119 (31.4)
Females	257 (67.8)
Not reporting	3 (0.8)
Married or relationship	199 (52.5)
Neither	180 (47.5)
Age 25 and younger	130 (34.3)
26 to 34	128 (33.8)
35 and older	120 (31.7)
Not reporting	1 (0.3)
At or above median income*	186 (49.1)
Below median income	178 (47.0)
Not reporting	15 (4.0)
White	319 (84.2)
Non-White	60 (15.8)

Note, the median income was ₤50,000–₤54,000

Income was assessed within ₤5,000 bands

Data were collected online from May to August 2021, with recruitment taking place via advertisements on social media (e.g., LinkedIn, Facebook). Qualtrics© XM (Qualtrics, Provo UT, 2020) hosted the survey. Internet surveys are as reliable and valid as paper-and-pencil measures [28].

### PAI

The symptoms of generalized anxiety disorder [25] provided the basis for the ten symptom items on the PAI. The instrument asks respondents to report symptoms they experienced over the last month. Different from most standard psychological symptom scales, which present symptom items in a “cause-neutral” manner (e.g., the GAD-7), PAI items are worded such that they ask participants if they attribute any symptom to the pandemic (e.g., “I felt nervous or anxious or on edge because of the pandemic”). Asking respondents make causal attributions is common in clinical practice and research (e.g., acute stress disorder) [25] and in national surveys like the Stress in America™ survey [29]. The PAI is structured similarly. Items are rated on a 4-point scale, from 0 (“Never or almost never”) to 3 (“Nearly every day”). Scores were recoded to range from 1 to 4 (*M* = 1.731; *SD* = 0.601; alpha = 0.924; omega = 0.954). If participants experienced a symptom that they believed (a) was related to a difficulty other than the pandemic (e.g., marital problems) or (b) developed for an unknown reason, they were instructed to check 0. The PAI can be found in Supplement 1.

### Data analysis

We employed Mplus 8.7 [30] in a CFA to examine measurement invariance across genders, age groups, relationship statuses, income, and race (see Table 1), treating all PAI items as ordinal [31], and using the weighted least squares mean and variance adjusted estimator. Relationship status was defined as currently being married or in a relationship versus neither being married nor being in a relationship. As described in Table 1, three age groups were created for an evaluation of age-related invariance, each group comprising approximately one third of the sample. One participant who did not report his or her age was excluded. Income was reported categorically in terms of £5000 increments. Using a median split, we categorized those earning less than £50,000 as lower income and those earning £50,000 or more per year as higher income. We regarded the lower-income group as relatively disfavored. With regard to race/ethnicity, anyone who identified as White was so categorized (*n* = 319); anyone who identified as having origins in Africa, the Caribbean, the Indian subcontinent, etc. was categorized as Nonwhite (*n* = 60). Although we regarded the analysis using the Nonwhite subgroup with caution owing to its small size and heterogeneity, we grouped these individuals together reasoning that such a grouping provided us with a crude way to assess invariance across favored and disfavored groups.

We examined configural, metric, and scalar invariance. We examined changes in the comparative fit index (CFI) or ΔCFI, and changes in the standardized root mean square residual (SRMR) or ΔSRMR. We defined deviations from standard measurement invariance as follows: ΔCFI of at least -0.010 and ΔSRMR of at least 0.005 [11, 31, 32]. The constraints imposed by the less stringent type of invariance, for example configural, are imposed on the more stringent type of invariance, for example, metric. For the analysis of race/ethnicity, we merged categories 3 and 4 for PAI item 7 because of an empty cell in the Nonwhite group. With an empty cell Mplus will not work because the weights for weighted least squares estimation are defined by the inverse of the cell counts.

In Supplement 2, we show an analysis that complements the abovementioned analyses. In the supplemental analysis, we use the Multiple Indicator Multiple Causes (MIMIC) approach to invariance testing [33]. The MIMIC approach addresses scalar invariance, the most stringent type of invariance we assessed.

## Results

All measurement invariance findings are presented in Table 2. Regarding gender, the fit for configural invariance model was satisfactory. The CFI decreased marginally for metric and scalar invariance and the SRMR increased slightly. Regarding the three age groups, the fit for configural invariance model was satisfactory. The CFI was unchanged for metric invariance and increased slightly for scalar invariance. The SRMR increased slightly for metric and scalar invariance.

**Table 2:** Measurement invariance models

	*χ*^*2*^	*df*	CFI	ΔCFI	SRMR	ΔSRMR
Gender						
Configural model	138.489	70	0.991	―	0.040	―
Metric model	179.975	79	0.986	-0.005	0.044	0.004
Scalar model	201.550	98	0.986	0.000	0.046	0.002
Age groups						
Configural model	206.950	105	0.988	―	0.047	―
Metric model	227.619	123	0.988	0.000	0.049	0.002
Scalar model	245.105	161	0.990	0.002	0.051	0.002
Married/relationship status						
Configural model	140.726	70	0.992	―	0.037	―
Metric model	150.582	79	0.992	0.000	0.038	0.001
Scalar model	144.950	98	0.995	0.003	0.039	0.001
Income level						
Configural model	143.955	70	0.990	―	0.040	―
Metric model	151.192	79	0.991	0.001	0.040	0.000
Scalar model	153.522	98	0.993	0.002	0.041	0.001
Race/ethnicity						
Configural model	134.713	70	0.992	―	0.041	―
Metric model	137.411	79	0.993	0.001	0.041	0.000
Scalar model	145.279	97	0.994	0.001	0.043	0.002

The gender-related analyses involved 257 women and 119 men. Data on gender were missing from three participants. The age-related analyses involved three groups, 130 individuals ages 25 and under; 128, between the ages of 26 and 34; and 120 ages 35 and older. Data on age were missing for one participant. The relationship analyses involved 199 participants who were married or in a relationship and 180 who were neither. The analyses related to income involved 178 participants who earned £49,000 or less and 186, who earned £50,000 or more. Fifteen individuals did not report income. The analyses bearing on race/ethnicity involved 319 White participants and 60, Non-White participants

*CFI* Comparative fit index, *ΔCFI* Delta (change in) CFI, *SRMR* Standardized root mean squared residual, *ΔSRMR* Change in SRMR, *df* Degrees of freedom

The fit for the configural invariance model across relationship statuses was satisfactory. The CFI remained the same or increased slightly for the metric and scalar models. The SRMR increased slightly. We found a satisfactory fit for the configural invariance model across income levels. The CFI increased slightly for the metric and scalar models. The SRMR remained the same for metric invariance and increased slightly for scalar invariance. Finally, the fit for configural invariance model across racial/ethnic groups was satisfactory. The CFI increased slightly for metric and scalar invariance. The SRMR remained the same for metric invariance increased slightly for scalar invariance. The MIMIC analysis described in Supplement 2 underlines scalar invariance related to group membership.

## Discussion

The PAI demonstrated measurement invariance across the demographic groups under scrutiny, consistent with the idea that the PAI assesses the same construct across those groups. The PAI behaved similarly across genders, age groups, and relationship statuses, as well as across favored and disfavored groups as per the analyses applied to income levels and race/ethnicity. The MIMIC analysis was consistent with the abovementioned analyses. The findings are encouraging, suggesting the PAI has equivalent meaning among members of different demographic groups and PAI scores can be compared across these groups.

The quality of measures provides the foundation needed for clinical and research applications. Hussey and Hughes [34] found that psychological scales can fall apart when examined via rigorous validity assessments beyond the near-universally present calculation of the coefficient alpha. They observed that only 4% of several well-known self-report personality and social psychology scales showed evidence of measurement invariance. We constructed the PAI for the purpose of advancing clinical practice and research bearing on the psychological sequelae of pandemics. Between this study of measurement invariance among demographic groups and the previous study of the criterion and construct validity of the instrument [1], the PAI appears to be a promising instrument that may be helpful in research and practice.

Nevertheless, this study of the PAI has several limitations. First, a convenience sample was used. Moreover, the sample overrepresented individuals who had at least some higher education. It would be helpful if future researchers were to include higher numbers of individuals having less education. Second, the study was conducted in only one country. The third limitation is a corollary to the second. The Nonwhite group was relatively small (*n* = 60) and heterogeneous, our having grouped together members of different ethnicities, given the sample size. Research involving English-speaking (e.g., the United States) and non-English-speaking countries (e.g., Brazil) with large multi-ethnic populations would be helpful. Fourth, our data were cross-sectional, preventing an assessment of temporal measurement invariance.

That the PAI shows evidence of measurement invariance among various demographic groups complements and sustains previous findings [1] supporting the instrument’s construct validity. For example, the invariance findings shown in this paper reinforce previous between-group findings revealing that, in the context of the COVID pandemic, elevated PAI scores were related to increased financial strain, job loss, increased economic insecurity, the hospitalization of a close friend or loved one, the death of a close friend or loved one, other traumatic events, and the experience of the COVID pandemic leading the individual to consider a major life change, further underscoring the PAI’s promise [1]. The invariance findings also reinforce results that showed that scores on the PAI were related to poorer sleep, subjective estimates of how widespread the pandemic was in respondents’ localities, and reduced social support. Future research calls for trying out the instrument in English-speaking countries outside the United Kingdom. Translations into other languages would help in understanding the behavior of the PAI in other linguistic/cultural groups.

### Supplementary Information

Supplementary Material 1.

Supplementary Material 2.

## Data Availability

The raw data for this study are available from the corresponding author on reasonable request.

## References

[1] Schonfeld IS, Prytherch T, Cropley M, Bianchi R (2023). The Pandemic Anxiety Inventory: a validation study. J Hlth Psychol.

[2] Spitzer RL, Kroenke K, Williams JBW, Löwe B (2006). A brief measure for assessing generalized anxiety disorder: the GAD-7. Arch Intern Med.

[3] Bueno-Notivol J, Gracia-García P, Olaya B, Lasheras I, López-Antón R, Santabárbara J (2021). Prevalence of depression during the COVID-19 outbreak: a meta-analysis of community-based studies. Int J Clin Hlth Psychol..

[4] Carmassi C, Foghi C, Dell’Oste V, Cordone A, Bertelloni CA, Bui E, Dell’Osso L (2020). PTSD symptoms in healthcare workers facing the three coronavirus outbreaks: What we can expect after the COVID-19 pandemic. Psychiat Res.

[5] Targa ADS, Benítez ID, Moncusí-Moix A, Arguimbau M, de Batlle J, Dalmases M, Barbé F (2021). Decrease in sleep quality during COVID-19 outbreak. Sleep Breath.

[6] Delpino FM, da Silva CN, Jerônimo JS (2022). Prevalence of anxiety during the COVID-19 pandemic: a systematic review and meta-analysis of over 2 million people. J Affect Disord.

[7] Jafari P, Nozari F, Ahrari F, Bagheri Z (2017). Measurement invariance of the Depression Anxiety Stress Scales-21 across medical student genders. Int J Med Educ.

[8] Coulacoglou C, Saklofske DH. Advances in latent variable measurement modeling. In: Coulacoglou C, Saklofske DH, eds. Psychometrics and psychological assessment. Academic Press; 2017:67–88. 10.1016/B978-0-12-802219-1.00004-3.

[9] Hinz A, Sander C, Glaesmer H (2017). Optimism and pessimism in the general population: Psychometric properties of the Life Orientation Test (LOT-R). Int J Clin Hlth Psychol.

[10] Liu W, Lei H, Li L, Yi J (2015). Factorial invariance of the mood and anxiety symptom questionnaire-short form across gender. Pers Individ Dif.

[11] Putnick DL, Bornstein MH (2016). Measurement invariance conventions and reporting: The state of the art and future directions for psychological research. Dev Rev.

[12] Gomez R, Summers M, Summers A (2014). Depression anxiety stress scales-21: measurement and structural invariance across ratings of men and women. Assess.

[13] Norton PJ (2007). Depression Anxiety and Stress Scales (DASS-21): psychometric analysis across four racial groups. Anx Stress Coping.

[14] Osei TPS, Sawang S, Goh YW, Mukhtar F (2013). Using the Depression Anxiety Stress Scale 21 (DASS-21) across cultures. Int J Psychol.

[15] Lovibond SH, Lovibond P. Manual for the depression anxiety stress scales. Psychology Foundation Sydney, Australia; 1995.

[16] Talkovsky AM, Norton PJ (2015). the mood and anxiety symptom questionnaire across four ethnoracial groups in an undergraduate sample. Am J Orthopsychiat.

[17] Clark LA, Watson D (1991). Tripartite model of anxiety and depression: Psychometric evidence and taxonomic implications. J Abnorm Psychol.

[18] Nuevo R, Mackintosh M-A, Gatz M (2007). A test of the measurement invariance of a brief version of the Penn State Worry Questionnaire between American and Spanish older adults. Int Psychogeriat.

[19] Meyer TJ, Miller ML, Metzger RL, Borkovec TD (1990). Development and validation of the Penn State Worry Questionnaire. Behav Res Ther.

[20] Ober TM, Liu C, Cheng Y. Development, validation, and evidence of measurement invariance of a shortened measure of trait test anxiety. Euro J Psychol Assess. 2023. 10.1027/1015-5759/a000761.

[21] Moreno E, Muñoz-Navarro R, Medrano LA (2019). Factorial invariance of a computerized version of the GAD-7 across various demographic groups and over time in primary care patients. J Affect Disord.

[22] Stochl J, Fried EI, Fritz J (2022). On dimensionality, measurement invariance, and suitability of sum scores for the PHQ-9 and the GAD-7. Assess.

[23] Lee SA (2020). Coronavirus anxiety scale: a brief mental health screener for COVID-19 related anxiety. Death Stud.

[24] Sawicki AJ, Żemojtel-Piotrowska M, Balcerowska JM (2022). The fear of COVID-19 scale: Its structure and measurement invariance across 48 countries. Psychol Assess.

[25] American Psychiatric Association. Diagnostic and statistical manual of mental disorders (5th ed.). Washington: Author; 2013.

[26] Chan D. Advances in analytical strategies. In: S Zedeck, ed., APA handbook of industrial and organizational psychology (Vol. 1, pp. 85–113). American Psychological Association; 2011. 10.1037/12169-004.

[27] Bianchi R, Verkuilen J, Toker S, Schonfeld IS, Gerber M, Brähler E, Kroenke K (2022). Is the PHQ-9 a unidimensional measure of depression? A 58,272-participant study. Psychol Assess.

[28] Gosling SD, Mason W (2015). Internet research in psychology. Ann Rev Psychol.

[29] American Psychological Association. Stress in America™: Paying with our health. Author;2015.

[30] Muthén LK, Muthén BO. Mplus user’s guide (Version 8.7). Muthén & Muthén; 1998–2021.

[31] Shi D, Maydeu-Olivares A, Rosseel Y (2020). Assessing fit in ordinal factor analysis models: SRMR vs. RMSEA Struct Equat Modeling.

[32] Rutkowski L, Svetina D (2014). Assessing the hypothesis of measurement invariance in the context of large-scale international surveys. Educat Psychol Measurement.

[33] Muthén BO (1989). Latent variable modeling in heterogeneous populations. Psychometrika.

[34] Hussey I, Hughes S (2020). Hidden invalidity among 15 commonly used measures in social and personality psychology. Advances Meth Practices Psychol Sci.

