# Supplementary material for: Measurement invariance of the Pandemic Anxiety Inventory in different demographic groups
Source: BMC Psychol. 2024 Jun 17;12:353. doi: 10.1186/s40359-024-01829-z (PMC11181577; doi:10.1186/s40359-024-01829-z)
Supplement: Supplementary file 1 — Supplementary Material 1. [file 40359_2024_1829_MOESM1_ESM.docx]

PANDEMIC ANXIETY INVENTORY (PAI)

**PRELIMINARY INSTRUCTIONS TO RESPONDENTS**

The following statements concern the impact of the pandemic context could have had on you.

Please read each statement and indicate how often you experienced the problems mentioned over the PAST MONTH. Use the scale provided to respond:

**0** = never or almost never

**1** = a few days only

**2** = more days than not

**3** = nearly every day

Here is an example:

“I felt depressed because of the pandemic.”

• If you did NOT feel depressed because of the pandemic, select **0**.

• If you felt depressed for reasons that you consider UNCONNECTED TO THE PANDEMIC (personal problems, marital problems, family problems, health problems, etc.), select **0** as well.

• If you felt depressed but don’t know why, again select **0**.

• If it is clear for you that THE PANDEMIC caused you to feel depressed, select **1**, **2** or **3** to indicate how often that happened.

*You can now complete the questionnaire.*

**PANDEMIC ANXIETY INVENTORY (PAI)**

| Patient name: ……………………………………………………………………. | | Date: ………………………………………………. | | | |
| --- | --- | --- | --- | --- | --- |
| **Indicate how often you experienced the problems mentioned below over the past month.** | | *Never or almost never* | *A few days only* | *More days than not* | *Nearly every day* |
| 1. | I felt nervous or anxious or on edge because of the pandemic. | **0** | **1** | **2** | **3** |
| 2. | I was unable to stop or control the worrying caused by the pandemic. | **0** | **1** | **2** | **3** |
| 3. | The stress of the pandemic made me feel so restless that I had difficulty sitting still. | **0** | **1** | **2** | **3** |
| 4. | Independently of whether or not I was infected, the stress of experiencing the pandemic in my locality caused me to be easily fatigued. | **0** | **1** | **2** | **3** |
| 5. | I was so stressed about the pandemic that I experienced problems concentrating or memory lapses. | **0** | **1** | **2** | **3** |
| 6. | I was irritable or easily annoyed on account of the stress of the pandemic. | **0** | **1** | **2** | **3** |
| 7. | The pandemic stressed me so much that I experienced muscle tension or muscle pain. | **0** | **1** | **2** | **3** |
| 8. | Pandemic-related worries caused me to sleep poorly (for example: difficulty falling or staying asleep, waking up too early, unrestful sleep). | **0** | **1** | **2** | **3** |
| 9. | I was afraid that something awful might happen because of the pandemic (for example: getting infected, death of a close friend or relative, job loss, bankruptcy, public violence). | **0** | **1** | **2** | **3** |
| 10. | The experience of the pandemic was so stressful that I had trouble relaxing at home or at work. | **0** | **1** | **2** | **3** |
|  | **TOTAL SCORE:** | **…………………………………………………………...** | | | |

**If you have encountered at least some of the problems mentioned above, do these problems lead you to consider making major changes in your life (for example, leaving your current job or changing how you live at home)?**

□ Yes □ No □ I don’t know

**If you have encountered at least some of the problems mentioned above, to what extent did these problems affect your everyday functioning at home or at work?**

| □ Not at all | □ Mildly | □ Moderately | □ Strongly | □ Very strongly |
| --- | --- | --- | --- | --- |
