# Supplementary material for: Measurement invariance of the Pandemic Anxiety Inventory in different demographic groups
Source: BMC Psychol. 2024 Jun 17;12:353. doi: 10.1186/s40359-024-01829-z (PMC11181577; doi:10.1186/s40359-024-01829-z)
Supplement: Supplementary file 2 — Supplementary Material 2. [file 40359_2024_1829_MOESM2_ESM.docx]

**Supplemental Appendix: MIMIC Model Approach to Invariance Testing**

Muthén (1989)^1^ proposed the Multiple Indicator Multiple Causes (MIMIC) approach to invariance testing. This model is focused primarily on scalar invariance. While it is not as comprehensive as the multiple group approach insofar as it assumes some parameters are equivalent, it allows simultaneous testing of several variables, which is not practical with the multiple group approach. Because scalar invariance is the most important kind of invariance, we believe that the MIMIC approach is valuable. There are elaborations of it such as moderated nonlinear factor analysis (MNLFA) that enable testing all parameters, but the sample size requirements for it are not met by these data.^2^

The basic MIMIC model combines a CFA with a structural regression model relating covariates to the latent variable. The CFA model defines the latent variable, and the covariates are used to “explain” the latent variable, although the model is cross-sectional and should not be thought of as being causal, strictly speaking. In the MIMIC approach to invariance testing, additional paths extend from the covariates to the items that define the latent variable. When these paths are notable in magnitude, they indicate potential violations of scalar invariance.

Because MIMIC models with invariance involve many parameters, we made use of the penalized structural equation modeling (PSEM) framework,^3^ which adds penalty terms to selected parameters to improve identification and help prevent overfitting. For this specification, we used the recommended alignment penalty (ALF) with defaults that center the parameter estimate on 0 (We tried a number of variations of this model, including different penalties as well as some terms to deal with a few large residuals in the measurement model, but these did not have an appreciable effect, so they are not reported here.). Unfortunately, the resulting path diagram is too large to display, but in terms of the Mplus syntax, we used the following model:

MODEL:

!CFA component;

f by anx1 anx2 - anx10;

!regression of latent variable on covariates;

f on gender AgeTertile Relationship IncomeLevel WNW;

!regression of indicators on covariates;

anx1-anx9 on gender AgeTertile Relationship IncomeLevel WNW (m1-m45);

MODEL PRIORS:

!these are used to apply the ALF penalty term to the specified parameters;

!we experimented with other values, but there was no appreciable change;

m1-m45 ~ alf(0,10);

The full output is available below, but the most important thing to note is that the vast majority of regression coefficients relating the covariates to the items directly are small and non-significant. Only items 3 through 6 have statistically significant gender effects, indicating potential violation of scale invariance. However, these effects have small magnitude—fully standardized effect sizes of magnitude 0.25 or smaller. They are negative and smaller than the fully standardized coefficient for gender pointing in the positive direction on the latent variable. (These are highlighted in the output below.) This represents a fairly minor deviation from scalar invariance for gender; in sum, scalar invariance appears reasonably, if not perfectly, supported.

**References**

**_____________________**

^1^ Muthén BO. Latent variable modeling in heterogeneous populations. Psychometrika 2017; 54:557–585. <https://doi.org/10.1007/BF02296397>

^2^ Bauer DJ. A more general model for testing measurement invariance and differential item functioning. Psychol Methods 2017; 22(3):507-526. doi:10.1037/met0000077.

^3^ Asparouhov T, Muthén, B. Penalized structural equation models. Struct Equat Modeling, 2023. doi:10.1080/10705511.2023.2263913

Mplus VERSION 8.10

MUTHEN & MUTHEN

02/12/2024 6:41 PM

INPUT INSTRUCTIONS

TITLE:

!windows;

DATA:

FILE IS "C:\Users\jverk\Dropbox\Research\DBCFA\pandemic\outfiles\All_anxiety_items.dat";

!mac;

!DATA:

! FILE IS "C:/Users/jverk/Dropbox/Research/DBCFA/pandemic/All_anxiety_items.dat";

VARIABLE:

NAMES ARE ID Anx1 Anx2 Anx3 Anx4 Anx5 Anx6 Anx7 Anx8 Anx9 Anx10

gender AgeTertile Relationship IncomeLevel WNW;

USEVARIABLES ARE Anx1 Anx2 Anx3 Anx4 Anx5 Anx6 Anx7 Anx8 Anx9 Anx10

gender AgeTertile Relationship IncomeLevel WNW;

MISSING ARE ALL (-9);

CATEGORICAL ARE anx1 - anx10 ;

!CLASSES = g(2);

!KNOWNCLASS = g (incomelevel = 1 incomelevel = 2)

ANALYSIS:

TYPE IS general;

!LOGHIGH = +15;

!LOGLOW = -15;

!UCELLSIZE = 0.01;

ESTIMATOR IS wlsmv;

!LOGCRITERION = 0.0000001;

ITERATIONS = 1000;

CONVERGENCE = 0.000001;

!MITERATIONS = 500;

!MCONVERGENCE = 0.000001;

!MIXC = ITERATIONS;

!MCITERATIONS = 2;

!MIXU = ITERATIONS;

!MUITERATIONS = 2;

!alignment = fixed;

OUTPUT: stdyx residual;

MODEL:

f by anx1 anx2 - anx10;

f on gender AgeTertile Relationship IncomeLevel WNW;

anx1-anx10 on gender AgeTertile Relationship IncomeLevel WNW (m1-m50);

!excess local dependence;

!anx1 with anx5 (m46);

!anx7 with anx9 (m47);

MODEL PRIORS:

m1-m50 ~ alf(0,10);

*** WARNING in VARIABLE command

Note that only the first 8 characters of variable names are used in the output.

Shorten variable names to avoid any confusion.

*** WARNING

Data set contains cases with missing on x-variables.

These cases were not included in the analysis.

Number of cases with missing on x-variables: 18

2 WARNING(S) FOUND IN THE INPUT INSTRUCTIONS

SUMMARY OF ANALYSIS

Number of groups 1

Number of observations 361

Number of dependent variables 10

Number of independent variables 5

Number of continuous latent variables 1

Observed dependent variables

Binary and ordered categorical (ordinal)

ANX1 ANX2 ANX3 ANX4 ANX5 ANX6

ANX7 ANX8 ANX9 ANX10

Observed independent variables

GENDER AGETERTI RELATION INCOMELE WNW

Continuous latent variables

F

Estimator WLSMV

Maximum number of iterations 1000

Convergence criterion 0.100D-05

Maximum number of steepest descent iterations 20

Maximum number of iterations for H1 2000

Convergence criterion for H1 0.100D-03

Parameterization DELTA

Link PROBIT

Input data file(s)

C:\Users\jverk\Dropbox\Research\DBCFA\pandemic\outfiles\All_anxiety_items.dat

Input data format FREE

SUMMARY OF DATA

Number of missing data patterns 1

COVARIANCE COVERAGE OF DATA

Minimum covariance coverage value 0.100

PROPORTION OF DATA PRESENT

Covariance Coverage

ANX1 ANX2 ANX3 ANX4 ANX5

________ ________ ________ ________ ________

ANX1 1.000

ANX2 1.000 1.000

ANX3 1.000 1.000 1.000

ANX4 1.000 1.000 1.000 1.000

ANX5 1.000 1.000 1.000 1.000 1.000

ANX6 1.000 1.000 1.000 1.000 1.000

ANX7 1.000 1.000 1.000 1.000 1.000

ANX8 1.000 1.000 1.000 1.000 1.000

ANX9 1.000 1.000 1.000 1.000 1.000

ANX10 1.000 1.000 1.000 1.000 1.000

Covariance Coverage

ANX6 ANX7 ANX8 ANX9 ANX10

________ ________ ________ ________ ________

ANX6 1.000

ANX7 1.000 1.000

ANX8 1.000 1.000 1.000

ANX9 1.000 1.000 1.000 1.000

ANX10 1.000 1.000 1.000 1.000 1.000

UNIVARIATE PROPORTIONS AND COUNTS FOR CATEGORICAL VARIABLES

ANX1

Category 1 0.219 79.000

Category 2 0.532 192.000

Category 3 0.199 72.000

Category 4 0.050 18.000

ANX2

Category 1 0.471 170.000

Category 2 0.371 134.000

Category 3 0.133 48.000

Category 4 0.025 9.000

ANX3

Category 1 0.596 215.000

Category 2 0.343 124.000

Category 3 0.039 14.000

Category 4 0.022 8.000

ANX4

Category 1 0.413 149.000

Category 2 0.399 144.000

Category 3 0.150 54.000

Category 4 0.039 14.000

ANX5

Category 1 0.684 247.000

Category 2 0.238 86.000

Category 3 0.050 18.000

Category 4 0.028 10.000

ANX6

Category 1 0.324 117.000

Category 2 0.349 126.000

Category 3 0.274 99.000

Category 4 0.053 19.000

ANX7

Category 1 0.731 264.000

Category 2 0.230 83.000

Category 3 0.030 11.000

Category 4 0.008 3.000

ANX8

Category 1 0.429 155.000

Category 2 0.416 150.000

Category 3 0.133 48.000

Category 4 0.022 8.000

ANX9

Category 1 0.294 106.000

Category 2 0.488 176.000

Category 3 0.163 59.000

Category 4 0.055 20.000

ANX10

Category 1 0.488 176.000

Category 2 0.393 142.000

Category 3 0.083 30.000

Category 4 0.036 13.000

UNIVARIATE SAMPLE STATISTICS

UNIVARIATE HIGHER-ORDER MOMENT DESCRIPTIVE STATISTICS

Variable/ Mean/ Skewness/ Minimum/ % with Percentiles

Sample Size Variance Kurtosis Maximum Min/Max 20%/60% 40%/80% Median

GENDER 0.676 -0.752 0.000 32.41% 0.000 1.000 1.000

361.000 0.219 -1.435 1.000 67.59% 1.000 1.000

AGETERTILE 1.972 0.051 1.000 34.35% 1.000 2.000 2.000

361.000 0.659 -1.480 3.000 31.58% 2.000 3.000

RELATIONSHIP 0.529 -0.117 0.000 47.09% 0.000 0.000 1.000

361.000 0.249 -1.986 1.000 52.91% 1.000 1.000

INCOMELEVEL 1.510 -0.039 1.000 49.03% 1.000 1.000 2.000

361.000 0.250 -1.998 2.000 50.97% 2.000 2.000

WNW 0.161 1.848 0.000 83.93% 0.000 0.000 0.000

361.000 0.135 1.416 1.000 16.07% 0.000 0.000

THE MODEL ESTIMATION TERMINATED NORMALLY

MODEL FIT INFORMATION

Number of Free Parameters 90

Chi-Square Test of Model Fit

Value 98.653*

Degrees of Freedom 35

P-Value 0.0000

* The chi-square value for MLM, MLMV, MLR, ULSMV, WLSM and WLSMV cannot be used

for chi-square difference testing in the regular way. MLM, MLR and WLSM

chi-square difference testing is described on the Mplus website. MLMV, WLSMV,

and ULSMV difference testing is done using the DIFFTEST option.

RMSEA (Root Mean Square Error Of Approximation)

Estimate 0.064

90 Percent C.I. 0.048 0.080

Probability RMSEA <= .05 0.074

CFI/TLI

CFI 0.990

TLI 0.974

Chi-Square Test of Model Fit for the Baseline Model

Value 6759.227

Degrees of Freedom 95

P-Value 0.0000

SRMR (Standardized Root Mean Square Residual)

Value 0.023

Optimum Function Value for Weighted Least-Squares Estimator

Value 0.71595801D-01

MODEL RESULTS

Two-Tailed

Estimate S.E. Est./S.E. P-Value

F BY

ANX1 1.000 0.000 999.000 999.000

ANX2 1.002 0.029 34.628 0.000

ANX3 0.867 0.035 24.520 0.000

ANX4 0.946 0.028 33.599 0.000

ANX5 0.936 0.033 28.229 0.000

ANX6 0.882 0.033 26.909 0.000

ANX7 0.803 0.047 17.047 0.000

ANX8 0.946 0.028 33.213 0.000

ANX9 0.866 0.032 26.778 0.000

ANX10 0.984 0.029 34.177 0.000

F ON

GENDER 0.485 0.143 3.388 0.001

AGETERTILE -0.123 0.077 -1.594 0.111

RELATIONSH -0.086 0.150 -0.569 0.570

INCOMELEVE -0.094 0.136 -0.693 0.488

WNW 0.257 0.148 1.737 0.082

ANX1 ON

GENDER 0.151 0.128 1.185 0.236

AGETERTILE 0.005 0.046 0.103 0.918

RELATIONSH 0.028 0.114 0.243 0.808

INCOMELEVE 0.106 0.130 0.819 0.413

WNW 0.190 0.143 1.332 0.183

ANX2 ON

GENDER 0.212 0.133 1.593 0.111

AGETERTILE -0.014 0.046 -0.312 0.755

RELATIONSH 0.095 0.155 0.610 0.542

INCOMELEVE -0.060 0.132 -0.454 0.650

WNW 0.133 0.150 0.891 0.373

ANX3 ON

GENDER -0.377 0.129 -2.924 0.003

AGETERTILE -0.045 0.083 -0.550 0.583

RELATIONSH 0.031 0.128 0.244 0.807

INCOMELEVE -0.081 0.134 -0.601 0.548

WNW -0.017 0.121 -0.138 0.890

ANX4 ON

GENDER -0.221 0.127 -1.739 0.082

AGETERTILE 0.009 0.053 0.162 0.871

RELATIONSH -0.035 0.121 -0.288 0.774

INCOMELEVE 0.028 0.103 0.271 0.787

WNW 0.005 0.071 0.067 0.947

ANX5 ON

GENDER -0.215 0.131 -1.636 0.102

AGETERTILE -0.013 0.071 -0.188 0.851

RELATIONSH 0.091 0.169 0.535 0.592

INCOMELEVE 0.002 0.060 0.031 0.975

WNW -0.036 0.160 -0.225 0.822

ANX6 ON

GENDER -0.329 0.124 -2.662 0.008

AGETERTILE -0.089 0.061 -1.459 0.144

RELATIONSH -0.090 0.141 -0.638 0.524

INCOMELEVE 0.058 0.121 0.479 0.632

WNW -0.143 0.148 -0.965 0.334

ANX7 ON

GENDER -0.002 0.059 -0.039 0.969

AGETERTILE 0.257 0.097 2.650 0.008

RELATIONSH -0.137 0.198 -0.696 0.487

INCOMELEVE -0.146 0.162 -0.903 0.367

WNW 0.013 0.131 0.102 0.919

ANX8 ON

GENDER 0.022 0.087 0.250 0.803

AGETERTILE 0.191 0.070 2.738 0.006

RELATIONSH 0.009 0.035 0.254 0.800

INCOMELEVE -0.001 0.058 -0.009 0.993

WNW -0.095 0.136 -0.702 0.483

ANX9 ON

GENDER -0.020 0.104 -0.195 0.845

AGETERTILE 0.007 0.053 0.124 0.901

RELATIONSH -0.040 0.134 -0.299 0.765

INCOMELEVE 0.120 0.125 0.960 0.337

WNW 0.115 0.138 0.832 0.406

ANX10 ON

GENDER 0.180 0.114 1.576 0.115

AGETERTILE 0.087 0.072 1.207 0.228

RELATIONSH -0.164 0.135 -1.217 0.224

INCOMELEVE -0.060 0.112 -0.531 0.596

WNW 0.004 0.065 0.059 0.953

Thresholds

ANX1$1 -0.561 0.241 -2.332 0.020

ANX1$2 0.984 0.241 4.091 0.000

ANX1$3 2.007 0.243 8.258 0.000

ANX2$1 -0.046 0.251 -0.184 0.854

ANX2$2 1.115 0.255 4.364 0.000

ANX2$3 2.123 0.272 7.814 0.000

ANX3$1 -0.268 0.280 -0.956 0.339

ANX3$2 1.064 0.266 3.999 0.000

ANX3$3 1.544 0.285 5.409 0.000

ANX4$1 -0.389 0.252 -1.546 0.122

ANX4$2 0.737 0.250 2.943 0.003

ANX4$3 1.640 0.262 6.269 0.000

ANX5$1 0.300 0.290 1.037 0.300

ANX5$2 1.256 0.283 4.434 0.000

ANX5$3 1.764 0.293 6.013 0.000

ANX6$1 -0.907 0.239 -3.802 0.000

ANX6$2 0.023 0.236 0.096 0.923

ANX6$3 1.229 0.254 4.843 0.000

ANX7$1 0.798 0.302 2.646 0.008

ANX7$2 1.972 0.295 6.690 0.000

ANX7$3 2.597 0.332 7.823 0.000

ANX8$1 0.144 0.257 0.562 0.574

ANX8$2 1.367 0.253 5.413 0.000

ANX8$3 2.376 0.269 8.830 0.000

ANX9$1 -0.436 0.242 -1.798 0.072

ANX9$2 0.928 0.242 3.837 0.000

ANX9$3 1.772 0.243 7.288 0.000

ANX10$1 0.021 0.255 0.083 0.934

ANX10$2 1.310 0.252 5.190 0.000

ANX10$3 1.969 0.258 7.644 0.000

Residual Variances

F 0.771 0.032 23.888 0.000

QUALITY OF NUMERICAL RESULTS

Condition Number for the Information Matrix 0.273E-04

(ratio of smallest to largest eigenvalue)

Prior Proportion 0.547E-01

(ratio of prior to prior plus chi-square)

STANDARDIZED MODEL RESULTS

STDYX Standardization

Two-Tailed

Estimate S.E. Est./S.E. P-Value

F BY

ANX1 0.872 0.023 37.318 0.000

ANX2 0.864 0.027 32.135 0.000

ANX3 0.786 0.036 22.097 0.000

ANX4 0.857 0.026 33.164 0.000

ANX5 0.851 0.030 28.390 0.000

ANX6 0.795 0.030 26.080 0.000

ANX7 0.723 0.041 17.492 0.000

ANX8 0.853 0.025 34.766 0.000

ANX9 0.777 0.029 26.631 0.000

ANX10 0.856 0.026 32.457 0.000

F ON

GENDER 0.246 0.069 3.554 0.000

AGETERTILE -0.108 0.068 -1.600 0.110

RELATIONSH -0.046 0.081 -0.570 0.569

INCOMELEVE -0.051 0.073 -0.694 0.487

WNW 0.102 0.059 1.749 0.080

ANX1 ON

GENDER 0.067 0.056 1.192 0.233

AGETERTILE 0.004 0.035 0.103 0.918

RELATIONSH 0.013 0.054 0.243 0.808

INCOMELEVE 0.050 0.061 0.817 0.414

WNW 0.066 0.049 1.337 0.181

ANX2 ON

GENDER 0.093 0.057 1.614 0.106

AGETERTILE -0.011 0.035 -0.312 0.755

RELATIONSH 0.044 0.072 0.610 0.542

INCOMELEVE -0.028 0.061 -0.455 0.649

WNW 0.046 0.051 0.895 0.371

ANX3 ON

GENDER -0.173 0.060 -2.897 0.004

AGETERTILE -0.036 0.066 -0.551 0.582

RELATIONSH 0.015 0.063 0.244 0.807

INCOMELEVE -0.040 0.066 -0.603 0.547

WNW -0.006 0.044 -0.138 0.890

ANX4 ON

GENDER -0.101 0.059 -1.727 0.084

AGETERTILE 0.007 0.042 0.162 0.871

RELATIONSH -0.017 0.059 -0.288 0.774

INCOMELEVE 0.014 0.050 0.271 0.787

WNW 0.002 0.026 0.067 0.947

ANX5 ON

GENDER -0.099 0.061 -1.622 0.105

AGETERTILE -0.011 0.057 -0.189 0.850

RELATIONSH 0.044 0.083 0.535 0.593

INCOMELEVE 0.001 0.029 0.031 0.975

WNW -0.013 0.058 -0.224 0.822

ANX6 ON

GENDER -0.150 0.057 -2.634 0.008

AGETERTILE -0.071 0.048 -1.467 0.142

RELATIONSH -0.044 0.069 -0.639 0.523

INCOMELEVE 0.028 0.059 0.479 0.632

WNW -0.051 0.053 -0.964 0.335

ANX7 ON

GENDER -0.001 0.027 -0.039 0.969

AGETERTILE 0.204 0.076 2.682 0.007

RELATIONSH -0.067 0.096 -0.697 0.486

INCOMELEVE -0.071 0.078 -0.907 0.364

WNW 0.005 0.047 0.102 0.919

ANX8 ON

GENDER 0.010 0.040 0.250 0.803

AGETERTILE 0.151 0.055 2.734 0.006

RELATIONSH 0.004 0.017 0.254 0.800

INCOMELEVE 0.000 0.028 -0.009 0.993

WNW -0.034 0.049 -0.702 0.483

ANX9 ON

GENDER -0.009 0.047 -0.195 0.845

AGETERTILE 0.005 0.042 0.124 0.901

RELATIONSH -0.019 0.065 -0.299 0.765

INCOMELEVE 0.058 0.061 0.957 0.339

WNW 0.041 0.049 0.834 0.404

ANX10 ON

GENDER 0.079 0.050 1.591 0.112

AGETERTILE 0.066 0.055 1.207 0.228

RELATIONSH -0.077 0.063 -1.221 0.222

INCOMELEVE -0.028 0.053 -0.531 0.595

WNW 0.001 0.022 0.059 0.953

Thresholds

ANX1$1 -0.529 0.228 -2.322 0.020

ANX1$2 0.928 0.225 4.127 0.000

ANX1$3 1.892 0.226 8.372 0.000

ANX2$1 -0.043 0.234 -0.184 0.854

ANX2$2 1.039 0.237 4.378 0.000

ANX2$3 1.979 0.256 7.722 0.000

ANX3$1 -0.263 0.272 -0.964 0.335

ANX3$2 1.043 0.269 3.874 0.000

ANX3$3 1.514 0.290 5.221 0.000

ANX4$1 -0.381 0.246 -1.550 0.121

ANX4$2 0.722 0.247 2.928 0.003

ANX4$3 1.606 0.258 6.219 0.000

ANX5$1 0.295 0.286 1.034 0.301

ANX5$2 1.235 0.282 4.381 0.000

ANX5$3 1.734 0.291 5.960 0.000

ANX6$1 -0.883 0.228 -3.874 0.000

ANX6$2 0.022 0.230 0.096 0.924

ANX6$3 1.197 0.253 4.740 0.000

ANX7$1 0.777 0.291 2.668 0.008

ANX7$2 1.919 0.283 6.789 0.000

ANX7$3 2.527 0.321 7.878 0.000

ANX8$1 0.141 0.250 0.564 0.573

ANX8$2 1.333 0.241 5.531 0.000

ANX8$3 2.318 0.256 9.055 0.000

ANX9$1 -0.423 0.235 -1.796 0.073

ANX9$2 0.900 0.234 3.849 0.000

ANX9$3 1.719 0.235 7.301 0.000

ANX10$1 0.020 0.240 0.083 0.934

ANX10$2 1.232 0.236 5.220 0.000

ANX10$3 1.852 0.240 7.719 0.000

Residual Variances

F 0.901 0.039 22.863 0.000

R-SQUARE

Observed Two-Tailed Residual

Variable Estimate S.E. Est./S.E. P-Value Variance

ANX1 0.796 0.030 26.916 0.000 0.229

ANX2 0.804 0.030 26.362 0.000 0.226

ANX3 0.596 0.042 14.150 0.000 0.420

ANX4 0.703 0.035 20.208 0.000 0.310

ANX5 0.686 0.041 16.629 0.000 0.325

ANX6 0.620 0.038 16.149 0.000 0.400

ANX7 0.524 0.056 9.377 0.000 0.503

ANX8 0.705 0.035 20.373 0.000 0.310

ANX9 0.603 0.039 15.353 0.000 0.422

ANX10 0.776 0.033 23.757 0.000 0.254

Latent Two-Tailed

Variable Estimate S.E. Est./S.E. P-Value

F 0.099 0.039 2.502 0.012

RESIDUAL OUTPUT

ESTIMATED MODEL AND RESIDUALS (OBSERVED - ESTIMATED)

Model Estimated Means/Intercepts/Thresholds

ANX1$1 ANX1$2 ANX1$3 ANX2$1 ANX2$2

________ ________ ________ ________ ________

-0.561 0.984 2.007 -0.046 1.115

Model Estimated Means/Intercepts/Thresholds

ANX2$3 ANX3$1 ANX3$2 ANX3$3 ANX4$1

________ ________ ________ ________ ________

2.123 -0.268 1.064 1.544 -0.389

Model Estimated Means/Intercepts/Thresholds

ANX4$2 ANX4$3 ANX5$1 ANX5$2 ANX5$3

________ ________ ________ ________ ________

0.737 1.640 0.300 1.256 1.764

Model Estimated Means/Intercepts/Thresholds

ANX6$1 ANX6$2 ANX6$3 ANX7$1 ANX7$2

________ ________ ________ ________ ________

-0.907 0.023 1.229 0.798 1.972

Model Estimated Means/Intercepts/Thresholds

ANX7$3 ANX8$1 ANX8$2 ANX8$3 ANX9$1

________ ________ ________ ________ ________

2.597 0.144 1.367 2.376 -0.436

Model Estimated Means/Intercepts/Thresholds

ANX9$2 ANX9$3 ANX10$1 ANX10$2 ANX10$3

________ ________ ________ ________ ________

0.928 1.772 0.021 1.310 1.969

Residuals for Means/Intercepts/Thresholds

ANX1$1 ANX1$2 ANX1$3 ANX2$1 ANX2$2

________ ________ ________ ________ ________

0.000 0.000 0.000 0.000 0.000

Residuals for Means/Intercepts/Thresholds

ANX2$3 ANX3$1 ANX3$2 ANX3$3 ANX4$1

________ ________ ________ ________ ________

0.000 0.000 0.000 0.000 0.000

Residuals for Means/Intercepts/Thresholds

ANX4$2 ANX4$3 ANX5$1 ANX5$2 ANX5$3

________ ________ ________ ________ ________

0.000 0.000 0.000 0.000 0.000

Residuals for Means/Intercepts/Thresholds

ANX6$1 ANX6$2 ANX6$3 ANX7$1 ANX7$2

________ ________ ________ ________ ________

0.000 0.000 0.000 0.000 0.000

Residuals for Means/Intercepts/Thresholds

ANX7$3 ANX8$1 ANX8$2 ANX8$3 ANX9$1

________ ________ ________ ________ ________

0.000 0.000 0.000 0.000 0.000

Residuals for Means/Intercepts/Thresholds

ANX9$2 ANX9$3 ANX10$1 ANX10$2 ANX10$3

________ ________ ________ ________ ________

0.000 0.000 0.000 0.000 0.000

Model Estimated Slopes

GENDER AGETERTI RELATION INCOMELE WNW

________ ________ ________ ________ ________

ANX1 0.636 -0.119 -0.058 0.012 0.448

ANX2 0.697 -0.138 0.009 -0.154 0.391

ANX3 0.043 -0.152 -0.043 -0.162 0.207

ANX4 0.238 -0.108 -0.116 -0.061 0.248

ANX5 0.239 -0.129 0.010 -0.086 0.205

ANX6 0.098 -0.198 -0.165 -0.025 0.084

ANX7 0.387 0.158 -0.206 -0.221 0.220

ANX8 0.480 0.074 -0.072 -0.089 0.148

ANX9 0.400 -0.100 -0.114 0.038 0.338

ANX10 0.657 -0.034 -0.248 -0.152 0.257

Residuals for Slopes

GENDER AGETERTI RELATION INCOMELE WNW

________ ________ ________ ________ ________

ANX1 0.002 0.000 0.003 0.002 0.003

ANX2 0.002 -0.001 0.004 -0.003 0.004

ANX3 -0.002 -0.002 0.004 -0.003 -0.004

ANX4 -0.002 0.001 -0.004 0.003 0.001

ANX5 -0.002 -0.001 0.005 0.000 -0.006

ANX6 -0.001 -0.001 -0.003 0.003 -0.003

ANX7 0.000 0.001 -0.005 -0.003 0.005

ANX8 0.003 0.001 0.002 0.000 -0.004

ANX9 -0.002 0.000 -0.004 0.002 0.004

ANX10 0.002 0.002 -0.003 -0.003 0.001

Model Estimated Covariances/Correlations/Residual Correlations

ANX1 ANX2 ANX3 ANX4 ANX5

________ ________ ________ ________ ________

ANX1

ANX2 0.773

ANX3 0.669 0.670

ANX4 0.729 0.731 0.633

ANX5 0.722 0.723 0.626 0.683

ANX6 0.680 0.682 0.590 0.643 0.637

ANX7 0.619 0.621 0.537 0.586 0.580

ANX8 0.729 0.731 0.633 0.690 0.683

ANX9 0.668 0.669 0.579 0.632 0.625

ANX10 0.758 0.760 0.658 0.718 0.710

Model Estimated Covariances/Correlations/Residual Correlations

ANX6 ANX7 ANX8 ANX9 ANX10

________ ________ ________ ________ ________

ANX7 0.546

ANX8 0.643 0.586

ANX9 0.589 0.536 0.632

ANX10 0.669 0.609 0.718 0.657

Residuals for Covariances/Correlations/Residual Correlations

ANX1 ANX2 ANX3 ANX4 ANX5

________ ________ ________ ________ ________

ANX1

ANX2 0.065

ANX3 -0.045 -0.009

ANX4 -0.060 -0.069 0.034

ANX5 -0.112 0.023 0.029 0.016

ANX6 0.032 -0.046 0.006 0.047 -0.008

ANX7 -0.089 -0.054 0.024 -0.002 0.061

ANX8 -0.023 -0.086 -0.032 0.032 0.015

ANX9 0.031 0.025 -0.013 -0.006 -0.044

ANX10 -0.012 -0.004 0.005 0.002 -0.003

Residuals for Covariances/Correlations/Residual Correlations

ANX6 ANX7 ANX8 ANX9 ANX10

________ ________ ________ ________ ________

ANX7 -0.004

ANX8 0.006 0.080

ANX9 -0.012 -0.121 -0.010

ANX10 -0.058 0.019 0.020 0.023

ESTIMATED MODEL AND RESIDUALS (OBSERVED - ESTIMATED) FOR THE JOINT MODEL

Model Estimated Means/Thresholds for the Joint Model (Dependent and Independent Variables)

ANX1$1 ANX1$2 ANX1$3 ANX2$1 ANX2$2

________ ________ ________ ________ ________

-0.817 0.728 1.751 -0.081 1.080

Model Estimated Means/Thresholds for the Joint Model (Dependent and Independent Variables)

ANX2$3 ANX3$1 ANX3$2 ANX3$3 ANX4$1

________ ________ ________ ________ ________

2.088 0.238 1.570 2.050 -0.223

Model Estimated Means/Thresholds for the Joint Model (Dependent and Independent Variables)

ANX4$2 ANX4$3 ANX5$1 ANX5$2 ANX5$3

________ ________ ________ ________ ________

0.903 1.806 0.485 1.440 1.948

Model Estimated Means/Thresholds for the Joint Model (Dependent and Independent Variables)

ANX6$1 ANX6$2 ANX6$3 ANX7$1 ANX7$2

________ ________ ________ ________ ________

-0.471 0.458 1.665 0.633 1.806

Model Estimated Means/Thresholds for the Joint Model (Dependent and Independent Variables)

ANX7$3 ANX8$1 ANX8$2 ANX8$3 ANX9$1

________ ________ ________ ________ ________

2.431 -0.177 1.046 2.055 -0.560

Model Estimated Means/Thresholds for the Joint Model (Dependent and Independent Variables)

ANX9$2 ANX9$3 ANX10$1 ANX10$2 ANX10$3

________ ________ ________ ________ ________

0.804 1.648 -0.035 1.253 1.913

Model Estimated Means/Thresholds for the Joint Model (Dependent and Independent Variables)

GENDER AGETERTI RELATION INCOMELE WNW

________ ________ ________ ________ ________

0.676 1.972 0.529 1.510 0.161

Residuals for Means/Thresholds for the Joint Model (Dependent and Independent Variables)

ANX1$1 ANX1$2 ANX1$3 ANX2$1 ANX2$2

________ ________ ________ ________ ________

-0.008 -0.008 -0.008 0.003 0.003

Residuals for Means/Thresholds for the Joint Model (Dependent and Independent Variables)

ANX2$3 ANX3$1 ANX3$2 ANX3$3 ANX4$1

________ ________ ________ ________ ________

0.003 0.008 0.008 0.008 -0.002

Residuals for Means/Thresholds for the Joint Model (Dependent and Independent Variables)

ANX4$2 ANX4$3 ANX5$1 ANX5$2 ANX5$3

________ ________ ________ ________ ________

-0.002 -0.002 0.002 0.002 0.002

Residuals for Means/Thresholds for the Joint Model (Dependent and Independent Variables)

ANX6$1 ANX6$2 ANX6$3 ANX7$1 ANX7$2

________ ________ ________ ________ ________

0.001 0.001 0.001 0.005 0.005

Residuals for Means/Thresholds for the Joint Model (Dependent and Independent Variables)

ANX7$3 ANX8$1 ANX8$2 ANX8$3 ANX9$1

________ ________ ________ ________ ________

0.005 -0.004 -0.004 -0.004 -0.001

Residuals for Means/Thresholds for the Joint Model (Dependent and Independent Variables)

ANX9$2 ANX9$3 ANX10$1 ANX10$2 ANX10$3

________ ________ ________ ________ ________

-0.001 -0.001 0.002 0.002 0.002

Residuals for Means/Thresholds for the Joint Model (Dependent and Independent Variables)

GENDER AGETERTI RELATION INCOMELE WNW

________ ________ ________ ________ ________

0.000 0.000 0.000 0.000 0.000

Model Estimated Covariances/Correlations for the Joint Model (Dependent and Independent Variables)

ANX1 ANX2 ANX3 ANX4 ANX5

________ ________ ________ ________ ________

ANX1 1.125

ANX2 0.906 1.151

ANX3 0.709 0.718 1.040

ANX4 0.793 0.801 0.669 1.042

ANX5 0.781 0.791 0.657 0.720 1.035

ANX6 0.729 0.736 0.632 0.685 0.671

ANX7 0.675 0.687 0.555 0.615 0.605

ANX8 0.797 0.807 0.643 0.718 0.710

ANX9 0.755 0.760 0.613 0.680 0.668

ANX10 0.877 0.894 0.704 0.784 0.770

GENDER 0.135 0.152 0.011 0.050 0.054

AGETERTI -0.124 -0.143 -0.132 -0.117 -0.106

RELATION -0.037 -0.037 -0.063 -0.059 -0.033

INCOMELE -0.021 -0.058 -0.064 -0.041 -0.037

WNW 0.051 0.042 0.032 0.034 0.025

Model Estimated Covariances/Correlations for the Joint Model (Dependent and Independent Variables)

ANX6 ANX7 ANX8 ANX9 ANX10

________ ________ ________ ________ ________

ANX6 1.054

ANX7 0.560 1.056

ANX8 0.655 0.632 1.051

ANX9 0.630 0.574 0.674 1.063

ANX10 0.724 0.679 0.789 0.740 1.130

GENDER 0.025 0.069 0.098 0.083 0.135

AGETERTI -0.174 0.015 0.000 -0.109 -0.126

RELATION -0.086 -0.036 -0.004 -0.047 -0.076

INCOMELE -0.045 -0.060 -0.023 -0.016 -0.068

WNW 0.017 0.028 0.012 0.042 0.030

Model Estimated Covariances/Correlations for the Joint Model (Dependent and Independent Variables)

GENDER AGETERTI RELATION INCOMELE WNW

________ ________ ________ ________ ________

GENDER 0.220

AGETERTI -0.045 0.660

RELATION 0.022 0.215 0.250

INCOMELE -0.001 0.111 0.093 0.251

WNW -0.020 -0.012 -0.027 -0.010 0.135

Residuals for Covariances/Correlations for the Joint Model (Dependent and Independent Variables)

ANX1 ANX2 ANX3 ANX4 ANX5

________ ________ ________ ________ ________

ANX1 0.000

ANX2 0.066 0.001

ANX3 -0.046 -0.009 0.000

ANX4 -0.060 -0.069 0.034 0.000

ANX5 -0.113 0.023 0.029 0.015 -0.001

ANX6 0.032 -0.046 0.006 0.047 -0.008

ANX7 -0.089 -0.053 0.024 -0.002 0.061

ANX8 -0.023 -0.086 -0.033 0.032 0.015

ANX9 0.031 0.026 -0.014 -0.006 -0.044

ANX10 -0.012 -0.003 0.005 0.002 -0.004

GENDER 0.000 0.001 0.000 -0.001 0.000

AGETERTI 0.001 0.000 0.000 0.000 0.000

RELATION 0.001 0.000 0.000 -0.001 0.001

INCOMELE 0.001 -0.001 -0.001 0.000 0.000

WNW 0.000 0.000 -0.001 0.000 -0.001

Residuals for Covariances/Correlations for the Joint Model (Dependent and Independent Variables)

ANX6 ANX7 ANX8 ANX9 ANX10

________ ________ ________ ________ ________

ANX6 0.000

ANX7 -0.004 0.001

ANX8 0.006 0.081 0.000

ANX9 -0.011 -0.121 -0.010 0.000

ANX10 -0.058 0.020 0.021 0.023 0.001

GENDER 0.000 0.000 0.001 -0.001 0.000

AGETERTI -0.001 -0.001 0.001 0.000 0.000

RELATION -0.001 -0.001 0.001 -0.001 -0.001

INCOMELE 0.000 -0.001 0.000 0.000 -0.001

WNW 0.000 0.001 -0.001 0.001 0.000

Residuals for Covariances/Correlations for the Joint Model (Dependent and Independent Variables)

GENDER AGETERTI RELATION INCOMELE WNW

________ ________ ________ ________ ________

GENDER 0.000

AGETERTI 0.000 0.000

RELATION 0.000 0.000 0.000

INCOMELE 0.000 0.000 0.000 0.000

WNW 0.000 0.000 0.000 0.000 0.000

Model Estimated Correlations for the Joint Model (Dependent and Independent Variables)

ANX1 ANX2 ANX3 ANX4 ANX5

________ ________ ________ ________ ________

ANX1 1.000

ANX2 0.797 1.000

ANX3 0.655 0.656 1.000

ANX4 0.732 0.731 0.642 1.000

ANX5 0.724 0.725 0.633 0.693 1.000

ANX6 0.669 0.668 0.604 0.654 0.643

ANX7 0.619 0.623 0.529 0.587 0.579

ANX8 0.733 0.734 0.615 0.686 0.681

ANX9 0.690 0.688 0.583 0.646 0.637

ANX10 0.777 0.784 0.649 0.722 0.712

GENDER 0.271 0.302 0.024 0.104 0.114

AGETERTI -0.143 -0.164 -0.160 -0.141 -0.128

RELATION -0.069 -0.069 -0.124 -0.116 -0.066

INCOMELE -0.039 -0.107 -0.125 -0.080 -0.073

WNW 0.130 0.106 0.085 0.090 0.067

Model Estimated Correlations for the Joint Model (Dependent and Independent Variables)

ANX6 ANX7 ANX8 ANX9 ANX10

________ ________ ________ ________ ________

ANX6 1.000

ANX7 0.531 1.000

ANX8 0.623 0.600 1.000

ANX9 0.596 0.542 0.638 1.000

ANX10 0.663 0.622 0.724 0.675 1.000

GENDER 0.052 0.144 0.203 0.172 0.272

AGETERTI -0.209 0.018 0.000 -0.130 -0.146

RELATION -0.168 -0.069 -0.008 -0.091 -0.143

INCOMELE -0.087 -0.116 -0.045 -0.031 -0.128

WNW 0.044 0.074 0.033 0.110 0.077

Model Estimated Correlations for the Joint Model (Dependent and Independent Variables)

GENDER AGETERTI RELATION INCOMELE WNW

________ ________ ________ ________ ________

GENDER 1.000

AGETERTI -0.118 1.000

RELATION 0.094 0.529 1.000

INCOMELE -0.004 0.274 0.374 1.000

WNW -0.116 -0.041 -0.146 -0.054 1.000

Residuals for Correlations for the Joint Model (Dependent and Independent Variables)

ANX1 ANX2 ANX3 ANX4 ANX5

________ ________ ________ ________ ________

ANX1 0.000

ANX2 0.057 0.000

ANX3 -0.042 -0.009 0.000

ANX4 -0.056 -0.063 0.032 0.000

ANX5 -0.104 0.021 0.028 0.015 0.000

ANX6 0.029 -0.042 0.006 0.045 -0.008

ANX7 -0.082 -0.049 0.023 -0.002 0.058

ANX8 -0.021 -0.079 -0.032 0.030 0.014

ANX9 0.028 0.023 -0.013 -0.006 -0.042

ANX10 -0.011 -0.003 0.004 0.001 -0.004

GENDER 0.001 0.001 0.000 -0.001 -0.001

AGETERTI 0.001 0.000 -0.001 0.000 0.000

RELATION 0.002 0.001 0.001 -0.001 0.002

INCOMELE 0.002 -0.001 -0.001 0.001 0.001

WNW 0.001 0.001 -0.001 0.001 -0.002

Residuals for Correlations for the Joint Model (Dependent and Independent Variables)

ANX6 ANX7 ANX8 ANX9 ANX10

________ ________ ________ ________ ________

ANX6 0.000

ANX7 -0.004 0.000

ANX8 0.005 0.076 0.000

ANX9 -0.011 -0.114 -0.010 0.000

ANX10 -0.054 0.017 0.018 0.021 0.000

GENDER -0.001 -0.001 0.001 -0.001 0.001

AGETERTI -0.001 -0.001 0.001 0.000 0.000

RELATION -0.001 -0.003 0.002 -0.002 -0.001

INCOMELE 0.001 -0.002 0.001 0.000 -0.002

WNW -0.001 0.002 -0.002 0.002 0.000

Residuals for Correlations for the Joint Model (Dependent and Independent Variables)

GENDER AGETERTI RELATION INCOMELE WNW

________ ________ ________ ________ ________

GENDER 0.000

AGETERTI 0.000 0.000

RELATION 0.000 0.000 0.000

INCOMELE 0.000 0.000 0.000 0.000

WNW 0.000 0.000 0.000 0.000 0.000

DIAGRAM INFORMATION

Use View Diagram under the Diagram menu in the Mplus Editor to view the diagram.

If running Mplus from the Mplus Diagrammer, the diagram opens automatically.

Diagram output

c:\users\jverk\dropbox\research\dbcfa\pandemic\mimic_alf010.dgm

Beginning Time: 18:41:05

Ending Time: 18:41:05

Elapsed Time: 00:00:00

MUTHEN & MUTHEN

3463 Stoner Ave.

Los Angeles, CA 90066

Tel: (310) 391-9971

Fax: (310) 391-8971

Web: www.StatModel.com

Support: Support@StatModel.com

Copyright (c) 1998-2023 Muthen & Muthen
